# Prenatal testing and prevalence of HIV infection during pregnancy: data from the “Birth in Brazil” study, a national hospital-based study

**DOI:** 10.1186/s12879-015-0837-8

**Published:** 2015-02-26

**Authors:** Rosa Maria Soares Madeira Domingues, Celia Landmann Szwarcwald, Paulo Roberto Borges Souza, Maria do Carmo Leal

**Affiliations:** Instituto Nacional de Infectologia Evandro Chagas (INI/Fiocruz), Av. Brasil, 4365-Manguinhos, Rio de Janeiro, CEP: 21040-360 Brasil; Instituto de Comunicação e Informação Científica e Tecnológica em Saúde (Icict/Fiocruz), Av. Brasil, 4.365 - Pavilhão Haity Moussatché–Manguinhos, Rio de Janeiro, CEP: 21045-360 Brasil; Departamento de Epidemiologia e Métodos Quantitativos em Saúde da Escola Nacional de Saúde Pública Sérgio Arouca/Fiocruz, Rua Leopoldo Bulhões, 1480 Manguinhos, Rio de Janeiro, Brasil

**Keywords:** HIV seroprevalence, Pregnancy, Prenatal care, Serological testing, Vertical transmission of infectious disease, Brazil

## Abstract

**Background:**

The rate of vertical HIV transmission has decreased in Brazil, but regional inequalities suggest problems in implementing control measures during pregnancy and delivery. The aims of this study were to ascertain the coverage of HIV testing during prenatal care and estimate the prevalence of HIV infection during pregnancy in Brazil.

**Methods:**

This was a national hospital-based study of 23,894 women that was conducted in 2011–2012. The data came from interviews with mothers during postpartum hospitalization, from hospital medical files and from prenatal cards. All the pregnant women with reactive serological results for HIV infection marked on their cards or with diagnoses of HIV infection during the hospital stay for delivery were considered cases of HIV infection. Univariate and multivariable logistic regression were performed to investigate factors associated with the prevalence of HIV infection and with performing at least one HIV test during pregnancy.

**Results:**

Among participating women, the coverage of testing for HIV infection was 81.7% among those who presented with prenatal card and the prevalence of HIV infection among pregnant women was 0.4% (95% CI: 0.32-0.51%). In the adjusted analysis, there was higher coverage of testing among women living in the South and Southeast regions; of women aged 35 years and over; with greater schooling levels; who self-reported as white; with prenatal care provided in private services; with an early start to prenatal care; and with an adequate number of consultations, defined as a minimum of six for a term pregnancy. In the adjusted analyses there was a greater odds ratio of HIV infection among women living in the South region, aged 35 years and over, with schooling of less than 8 years, who self-reported race as black, without a partner, with syphilis coinfection and who were attended by public services.

**Conclusions:**

The prevalence of HIV infection among pregnant women in Brazil remains below 1% and the coverage of testing for HIV infection is over 80%. However, the regional and social inequalities in access to healthcare services and the missed opportunities for diagnoses of HIV infection indicate the importance of strengthening HIV infection control programs during pregnancy.

## Background

Vertical transmission of HIV from mother to fetus during pregnancy, delivery and the postpartum period can be avoided in almost all cases through existing interventions, such as use of antiretroviral therapy, elective cesarean section and avoidance of breastfeeding [1]. Nonetheless, it has been estimated in 2009 that, worldwide, 370,000 children become infected with HIV every year [2].

Brazil has a concentrated AIDS epidemic with a stable prevalence estimated to be less than 1% [3]. Increased heterosexual transmission has resulted in an increased number of cases among women, while vertical HIV transmission from mother to child during pregnancy and delivery accounts for almost all of the cases reported among children [3]. Based on the last Sentinel Parturient Studies, conducted in 2006 [4] and 2010 [3], it was estimated that approximately 0.4% of pregnant women in Brazil have HIV infection. Given the number of cases of HIV infection reported among pregnant women in 2012, it was inferred that HIV surveillance among pregnant women only reached 58.3% of expected cases, [3] thus indicating that there were some difficulties in diagnosing and/or reporting cases.

In Brazil, several government initiatives have made tests, medications, infant formula feeding and care routines available for managing HIV during pregnancy, delivery and the postpartum period since the end of the 1990s [5]. Studies with national coverage in Brazil have shown that reductions in vertical transmission started to occur from 1997 onwards [6-9]. The estimated vertical transmission rate was 6.8% in 2004, with a range from 13.4% in the North region to 4.3% in the Midwest region [10]. Local studies conducted in the Southeast region [11-14] have reported vertical transmission rates closer to 3%, while rates of less than or equal to 2% have been found in the Midwest and South regions [15-17]. In contrast, in the North and Northeast regions and in the state of São Paulo, local studies have found vertical transmission rates of 9.9% [18], 9.2% [19] and 6.0% [20], respectively.

Thus, despite advances, flaws in the Brazilian control program for HIV during pregnancy have prevented reaching vertical transmission rates of less than 2%, like the rates found in some developed countries such as the United Kingdom [21]. The Pan-American Health Organization (PAHO) has a target of eliminating vertical transmission of HIV from the Latin American and Caribbean region by 2015, which translates into reaching a vertical transmission rate of less than or equal to 2% and an incidence of cases of vertical transmission of HIV of less than or equal to 0.3 per 1,000 live births [22].

Identification of HIV-infected pregnant women is the first stage towards adopting the protocols available for both prevention of vertical transmission and treatment of women. Local studies [13,15,18,19,23,24] have indicated that the coverage of HIV testing has increased in all Brazilian regions. However, these results were from studies with municipal or state coverage while no studies with national coverage that could adequately provide estimates for the country have been conducted recently.

The aims of the present study were to ascertain the coverage of HIV testing during prenatal care, to evaluate the factors associated with performing at least one HIV testing during pregnancy and to estimate the prevalence of HIV infection among pregnant women in Brazil through a survey with national coverage that was conducted among puerperal women who underwent delivery in hospitals in 2011–2012.

## Methods

The study “Birth in Brazil: national survey into labour and birth” was a hospital-based study conducted between February 2011 and October 2012. Additional information on the methodology of the study “Birth in Brazil” can be obtained from Leal et al. [25].

The sample was selected in three stages. In the first stage, hospitals with more than 500 deliveries per year were stratified according to the five macroregions of the country (North, Northeast, South, Southeast and Midwest), according to location (state capital or elsewhere in the state) and type of hospital service (public, mixed or private), and 266 hospitals were selected with a probability of selection proportional to the number of deliveries in each of the strata in 2007. The minimum size of the sample in each of the strata was 450 puerperal women. In the second stage, the number of days needed to interview 90 puerperal women in each hospital (minimum of 7) was selected by using an inverse sampling method. In the third stage, the women eligible on each day of the fieldwork were selected [26]. Sample losses due to refusal to participate or hospital discharge were replaced by selecting new puerperal women at the same hospital. In total, interviews were conducted with 23,894 women.

All puerperal women with live births in a hospital in which the fetus had any gestational age or weight, or a stillbirth in which the fetus had a gestational age of more than 22 weeks recorded in the medical file or weight greater than 500 grams, were considered eligible for the study. Miscarriages were excluded as the aim of the study was to evaluate the conditions of prenatal, birth and delivery care and the results from the care provided.

Interviews with the puerperal women were conducted during their hospital stay, at least 6 hours after delivery. Data from the medical files of the puerperal woman and newborn were obtained at the time of hospital discharge. In case of prolonged hospitalization, data from medical files were obtained at the 42nd day of hospitalization in the case of the puerperal women or the 28th day of life in the case of the newborns. For the interview and extraction of data from the medical files, electronic forms that had been developed specifically for this study were used. When available, prenatal cards, which are official forms provided by health services to all pregnant women during prenatal care where tests, procedures, and prescription drugs are recorded, were photographed in digital media with subsequent extraction and input of data into an online platform. This was done by a team of students and healthcare professionals who had been trained and were supervised by the central coordinating team of the study.

To evaluate the prenatal care, the protocols of the Ministry of Health were used. These recommend that prenatal care should begin by the 12^th^ gestational week [27], with a minimum of six consultations during follow-up. Furthermore, there should be two HIV serological tests over the course of the pregnancy: the first serological test should be requested at the time of the first prenatal visit and the second serological test should be requested at the start of the third trimester of the pregnancy [28].

In the present study, the following prenatal care indicators were estimated: proportion of puerperal women who reported having at least one prenatal consultation; proportion of puerperal women who reported having received a prenatal card; proportion of puerperal women who had their prenatal card at the time of hospital admission for delivery; and proportion of puerperal women with records of the first and second HIV tests on their card. It was assumed that the tests had been carried out when there was a record of any serological result relating to HIV. Other data on prenatal care that were analyzed in the “Birth in Brazil” study are available in Viellas et al. [29].

For the diagnosis of HIV infection during pregnancy, one of the following were considered: 1) reactive results recorded on the prenatal card (two rapid tests or Elisa + immunofluorescence or Elisa + Western Blot^a^); and/or 2) records in the hospital medical files of a) diagnosis of HIV infection or b) indication of cesarean due to HIV infection or c) use of zidovudine during delivery labor and/or delivery or d) use of zidovudine syrup for the newborn or e) suspension of breastfeeding due to maternal HIV infection or f) record of the diagnosis of “child exposed to HIV”.

The prenatal care indicators were analyzed according to the following maternal characteristics: “region of residence” (North, Northeast, Southeast, South or Midwest); “age” (12 to 19 years, 20 to 34 years or 35 years and over); “schooling level” (up to 7 years, 8 to 10 years or 11 or more years of school attendance); “self-reported skin color” (white, black, mixed, East Asian or indigenous); “conjugal situation” (with or without a partner); “type of prenatal care service” where most of the consultations were attended (public or private); and “type of delivery care service” (public, mixed or private; mixed services were taken to be those that attended to women with either public or private funding for delivery care). The chi-square statistical test was used to ascertain differences between proportions.

To evaluate the factors associated with performing at least one HIV testing during pregnancy, univariate and multivariable logistic regressions were performed. In addition to the maternal characteristics, the following variables relating to prenatal care were included: the time when prenatal care began measured in gestational weeks (up to week 12, between weeks 13 and 28, or week 29 and beyond); and the adequacy of the number of consultations up to the time of delivery, considering a minimum of six consultations for a term pregnancy, adjusted for the gestational age at birth (sufficient or insufficient), according to the criteria defined by the Ministry of Health.

To evaluate the factors associated with HIV infection among pregnant women, univariate and multivariable regressions were performed. In addition to the maternal characteristics described above, “diagnosis of syphilis infection during pregnancy” (yes or no) was also included, as some authors point to high rates of coinfection [30,31]. The women were considered to present with syphilis infection if they presented with one of the following criteria: record of a reactive serological test on the prenatal card or hospital medical file; record of syphilis diagnosis on the prenatal card or hospital medical file; or case of congenital syphilis as an outcome from the current pregnancy.

A univariate analysis estimated the unadjusted odds ratio (OR) and 95% confidence interval (95% CI). Variables with a significance level estimated as lower than 0.20 were included in an initial multivariable model and those with a significance level of <0.05 were retained in the final multivariable model. The results from the multivariable model were expressed as adjusted odds ratios with their corresponding 95% confidence intervals (95% CI).

In all the statistical analyses, the complex sampling design was taken into consideration. The data weighting was calculated according to the inverse of the probability of inclusion of each puerperal woman in the sample. To ensure that the distribution of the puerperal women interviewed was similar to that observed among the births in the population sampled in 2011, a calibration procedure was used in each selection stratum [26]. For the univariate and multivariable logistic regression, the women who self-reported as East Asian or indigenous were excluded because they accounted for a very small proportion of the sample. Analyses were performed using IBM SPSS software version 19.0 (IBM Corp., Armonk, USA).

This study was approved by the Research Ethics Committee of ENSP/Fiocruz, under report no. 92/2010. Every care was taken towards ensuring privacy and confidentiality of the information. Before each interview was conducted, the interviewee’s consent was obtained, after reading the free and informed consent statement.

## Results

Among the puerperal women selected to participate in the study, 5.7% were not interviewed either because they refused to participate or because they were discharged early. The majority of the puerperal women were living in the Southeast region (42.5%) and Northeast region (28.9%), which are the most populous regions of the country. The Midwest region had the lowest proportion (6.5%). The mean age of the women was 25.7 years, with a median of 25 years, and 19.1% were up to19 years of age. Half of the interviewees had attended school for up to 10 years and 8.9% had a college degree. The majority self-reported their skin color as mixed (56.1%), while East Asian and indigenous skin colors accounted for 1.1% and 0.4% of the sample total, respectively. More than 80% of the women said they had a partner. Nearly 99.0% of the puerperal women had had at least one prenatal consultation, 60.6% began their prenatal care early, i.e., up to the 12^th^ gestational week, and 71.6% had had sufficient consultations. More than 70% of the pregnant women had been attended in public clinics for their prenatal care, and only 14.5% had had their delivery in a private healthcare unit (Table 1).

**Table 1:** **Demographic and social characteristics and prenatal and delivery care service use, Brazil, 2011-2012**

**Maternal characteristics**	**n**	**%**
**Region of residence**		
Southeast	10.155	42.5
North	2.296	9.6
Northeast	6.904	28.9
South	2.984	12.5
Midwest	1.555	6.5
**Maternal age**		
12 to 19 years	4.570	19.1
20 to 34 years	16.807	70.4
35 and over	2.509	10.5
**Schooling level (years)**		
11 or more	11.371	47,8
8 to 10	6.085	25.6
0 to 7	6.322	26.6
**Skin color**		
White	8.079	33.8
Mixed	13.403	56.1
Black	2.051	8.6
East Asian	257	1.1
Indigenous	99	0.4
**Conjugal situation**		
With partner	19.439	81.4
Without partner	4.432	18.5
**Type of prenatal care service**		
No prenatal consultations	319	1.3
Public clinic	17.575	73.6
Private clinic	5.971	25.0
**Start of prenatal care**		
> week 28	857	3.7
Weeks 13 to 28	8.382	35.8
Up to week 12	14.182	60.6
**Adequacy of number of consultations** ^**1**^		
No	6.782	28.4
Yes	17.112	71.6
**Type of delivery care service**		
Private	3.462	14.5
Mixed	10.596	44.3
Public	9.836	41.2
**Total**	23.894	100

Totals for these variables vary because of missing values. For start of prenatal care and adequacy of number of consultations, total available for women who had prenatal care. ^1^Considering a minimum of six consultations for a term pregnancy, adjusted for gestational age at delivery.

Table 2 presents the prenatal care indicators: 96% of the puerperal women reported that they had received a prenatal card during their prenatal period and 71.6% presented with the card at the time of hospital admission for delivery. Of the analyzed cards, 81.7% had the results from the first HIV test recorded and 28.4% had the results from the second HIV test recorded.

**Table 2:** **Prenatal care and anti-HIV tests performed according to maternal and service characteristics, Brazil, 2011-2012**

**Maternal characteristics**	**n**	**Prenatal care (n = 23.894)**		**Received card (n = 23.555)**		**Presented card at maternity hospital (n = 23.555)**		**Result from one anti-HIV serological test (n = 16.899)**		**Results from two anti-HIV serological tests (n = 16.899)**	
		**%**	**p** ^**1**^	**%**	**p** ^**1**^	**%**	**p** ^**1**^	**%**	**p** ^**1**^	**%**	**p** ^**1**^
**Region of residence**											
Southeast	10.155	98.8	0.006	96.3	0.130	77.5	<0.001	88.2	<0.001	33.3	<0.001
North	2.296	97.5		97.9		64.2		69.9		18.4	
Northeast	6.904	98.5		94.9		67.9		68.4		15.1	
South	2.984	99.5		97.1		79.7		92.8		41.7	
Midwest	1.555	98.7		94.2		45.1		83.2		36.8	
**Maternal age**											
12 to 19 years	4.570	98.5	0.497	98.7	<0.001	74.7	<0.001	75.2	<0.001	26.0	0.020
20 to 34 years	16.807	98.7		95.8		71.7		83.0		28.7	
35 and over	2.509	98.7		92.4		65.9		85.8		31.0	
**Schooling level, (years)**											
11 or more	11.362	99.6	<0.001	93.4	<0.001	67.6	<0.001	88.2	<0.001	32.5	<0.001
8 to 10	6.081	98.8		98.0		74.5		80.8		28.5	
0 to 7	6.317	96.9		98.9		76.2		72.3		21.5	
**Skin color**											
White	8.079	99.2	0.001	94.2	<0.001	70.3	0.422	88.6	<0.001	34.6	<0.001
Mixed	13.403	98.5		96.8		72.1		78.3		25.3	
Black	2.051	98.1		98.4		73.4		77.1		24.5	
East Asian	257	98.3		93.5		73.9		77.7		27.0	
Indigenous	99	95.0		98.2		80.1		91.7		34.4	
**Conjugal situation**											
With partner	19.439	99.1		95.7		71.2		82.4		28.9	
Without partner	4.432	96.9	<0.001	97.4	0.002	73.4	0.107	78.7	0.001	26.1	0.023
**Type of prenatal service**											
Public clinic	17.575	---	---	99.3	<0.001	76.3	<0.001	79.3	<0.001	27.6	0.116
Private clinic	5.971	---	---	86.3		58.0		91.2		31.4	
**Type of delivery service**											
Public	9.836	97.8	<0.001	99.0	<0.001	73.9	<0.001	76.3	<0.001	24.8	0.036
Mixed	10.596	99.1		97.8		77.0		84.3		30.8	
Private	3.462	99.9		82.2		49.2		91.6		32.0	
**Total**	23.894	98.7		96.0		71.6		81.7		28.4	

^1^Chi-square statistical test.

Totals for these variables vary because of missing values. For type of prenatal service, total available for women who had prenatal care.

Fewer records of results from the first HIV test were observed in the North and Northeast regions; among women with a lower schooling level; among women who self-reported as black, mixed and East Asian; among those under 20 years of age; among those without a companion; and among those with prenatal and delivery care provided in public clinics. A similar pattern was observed for the results from the second HIV test, except in relation to the location where prenatal consultations took place: there was no difference between public and private clinics (Table 2).

In the multivariable analysis, having at least one HIV test during the pregnancy was associated with the region of residence, such that there was a greater odds of testing in the South region; with maternal age greater than or equal to 35 years; with prenatal care provided in private clinics; with an early start to prenatal care, particularly before the 12^th^ week of pregnancy; and with an adequate number of prenatal consultations. A lower odds of testing was observed for women who lived in the North and Northeast regions, who self-reported black or mixed and who had fewer years of schooling. No differences relating to conjugal situation were observed (Table 3).

**Table 3:** **Univariate and multivariable logistic regression on factors associated with performing one anti-HIV test, Brazil, 2011-2012**

**Maternal and prenatal care characteristics**	**n** ^**1**^	**One anti-HIV test performed during prenatal care (%)**	**Univariate analysis**			**Multivariable analysis** ^**2**^		
			**OR** ^**3**^	**95% CI** ^**4**^	**p**	**OR** ^**3**^	**95% CI** ^**4**^	**p**
**Region of residence**								
Southeast	7.783	88.2	1			1		
North	1.439	69.9	0.31	0.19-0.50	<0.001	0.47	0.30-0.76	<0.001
Northeast	4.616	68.4	0.29	0.21-0.40		0.38	0.28-0.52	
South	2.365	92.9	1.75	1.21-2.55		1.77	1.20-2.59	
Midwest	694	83.2	0.67	0.42-1.06		0.79	0.53-1.18	
**Maternal age**								
12 to 19 years	3.364	75.2	1		<0.001	1		
20 to 34 years	11.894	83.0	1.61	1.40-1.85		1.12	0.95-1.31	0.033
35 and over	1.633	85.8	2.00	1.64-2.46		1.33	1.07-1.64	
**Schooling level (years)**								
11 or more	7.658	88.2	1			1		
8 to 10	4.481	80.7	0.56	0.49-0.65	<0.001	0,78	0.68-0.90	<0.001
0 to 7	4.669	72.3	0.35	0.30-0.41		0,64	0.54-0.76	
**Skin color** ^**5**^								
White	5.634	88.6	1			1		
Mixed	9.520	78.3	0.43	0.34- 0.55	<0.001	0.77	0.67-0.90	0.001
Black	1.478	77.1	0.46	0.39- 0.53		0.73	0.57-0.94	
**Conjugal situation**								
With partner	13.724	82.4	1			1		
Without partner	3.155	78.7	0.79	0.68-0.91	0.001	0,93	0.79-1.09	0.345
**Prenatal service**								
Public	13.413	79.3	1		<0.001	1		
Private	3.462	91.2	2.49	2.02-3.07		1.47	1.17-1.86	0.001
**Start of prenatal care**								
>28^th^ week	747	59.3	1			1		
13^th^ to 28^th^ weeks	7.079	77.6	2.43	1.91-3.09	<0.001	1.88	1.39-2.54	<0.001
Up to 12^th^ week	9.020	87.0	4.68	3.66-5.99		2.34	1.70-3.21	
**Adequate number of consultations** ^**6**^								
No	4.756	66.7	1		<0.001	1		
Yes	12.141	87.5	3.45	2.99-3.99		2.17	1.87-2.52	<0.001

^1^Only women who attended antenatal care; ^2^All the analyses were adjusted for the following variables: region of residence, age, schooling level, skin color, conjugal situation, type of prenatal service, time when prenatal care started and adequacy of number of consultations for the gestational age; ^3^OR = odds ratio; ^4^CI = confidence interval; ^5^Pregnant women with East Asian or indigenous skin color were excluded from this analysis; ^6^Minimum of six consultations for a term pregnancy, adjusted for the gestational age at birth. Totals for these variables vary because of missing values.

Table 4 shows the prevalence of HIV infection among pregnant women, which was estimated to be 0.40% (95% CI: 0.32-0.51%). In the unadjusted analysis, a greater prevalence of infection was observed in the South than in the Southeast region. The prevalence increased with increasing maternal age and with decreasing schooling level. There was greater prevalence among women who self-reported as black, those without a companion, those with a diagnosis of syphilis during pregnancy and those whose delivery care was provided in public or mixed clinics.

**Table 4:** **Univariate and multivariable logistic regression on the maternal characteristics associated with HIV infection, Brazil, 2011-2012**

**Maternal characteristics**	**Prevalence of HIV infection (%)**	**95% CI**	**Univariate analysis**			**Multivariable analysis** ^**1**^		
			**OR** ^**2**^	**95% CI** ^**3**^	**p**	**OR** ^**2**^	**95% CI** ^**3**^	**p**
**Region of residence**								
Southeast	0.34	0.24-0.50	1			1		
North	0.44	0.18-1.09	1.30	0.49-3.47		1.11	0.43-2.90	
Northeast	0.29	0.15-0.57	0.82	0.38-1.75		0.70	0.31-1.58	
South	0.87	0.64-1.18	2.51	1.55-4.09	0.001	2.95	1.78-4.91	<0.001
Midwest	0.31	0.13-0.77	0.91	0.34-2.43		0.92	0.34-2.46	
**Maternal age**								
12 to 19 years	0.14	0.07-0.28	1			1		
20 to 34 years	0.44	0.34-0.58	3.04	1.47-6.30	0.004	4.45	2.08-9.51	<0.001
35 and over	0.60	0.35-1.04	4.17	1.75-9.98		6.33	2.54-15.80	
**Schooling level (years)**								
11 or more	0.24	0.16-0.35	1			1		
8 to 10	0.48	0.27-0.86	2.12	1.03-4.33		2.03	0.97-4.26	
0 to 7	0.63	0.46-0.87	2.78	1.62-4.78	0.001	2.26	1.28-3.98	0.014
**Skin color** ^**4**^								
White	0.32	0.23-0.46	1			1		
Mixed	0.35	0.25-0.48	1.08	0.67-1.74		1.09	0.67-1.79	
Black	1.09	0.65-1.82	3.38	1.78-6.39	<0.001	2.89	1.57-5.31	0.001
**Conjugal situation**								
With partner	0.35	0.26-0.47	1			1		
Without partner	0.63	0.44-0.90	1.83	1.18-2.86	0.008	1.87	1.21-2.87	0.005
**Syphilis infection** ^**5**^								
No	0.38	0.29-0.48	1			1		
Yes	2.86	1.20-6.70	7.92	3.05-20.58	<0.001	4.75	2.01-11.21	<0.001
**Type of delivery service** ^**6**^								
Private	0.10	0.04-0.22	1			1		
Mixed	0.33	0.22-0.50	3.40	1.38-8.38		2.11	0.83-5.34	
Public	0.58	0.43-0.79	5.94	2.53-13.96	<0.001	4.08	1.65-10.08	0.003

^1^All the analyses were adjusted for the following variables: region of residence, age, schooling level, skin color, conjugal situation, syphilis infection and type of delivery care service (public, mixed or private).

^2^OR = odds ratio; ^3^CI = confidence interval; ^4^Pregnant women with East Asian or indigenous skin color were excluded from this analysis.

^5^Women who presented with one of the following criteria were considered to present syphilis infection: record of a reactive serological test for syphilis on the prenatal card or the hospital medical files; record of a diagnosis of syphilis on the prenatal card or the hospital medical files; or a case of congenital syphilis as an outcome from the present pregnancy.

^6^Type of delivery service: public = deliveries only with public funding; private = deliveries only with private funding; mixed = deliveries with either public or private funding.

The results from the adjusted analysis (Table 4) showed that women living in the South region continued to have the greatest odds ratio of HIV infection (OR = 2.95; 95% CI 1.78-4.91). After adjustment for the other variables, the strength of association with the woman’s age increased, such that HIV infection among women aged 20 years or over was four to six times greater than that of adolescents. HIV infection was also associated with syphilis coinfection (OR = 4.75; 95% CI 2.01-11.21), with self-reported black skin color (OR = 2.89; 95% CI 1.57-5.31), with living without a partner (OR = 1.87; 95% CI 1.21-2.87), with less than 8 years of schooling (OR = 2.26; 95% CI 1.28-3.98) and with delivery care provided in public clinics (OR = 4.08; 95% CI 1.65-10.08). If the births that took place in the public and mixed clinics are analyzed together, the estimated prevalence was 0.45% (95% CI: 0.36-0.58%), a rate that was significantly higher than that observed in the private clinics (0.10%; 95% CI: 0.04-0.22), with an OR of 4.67 (95% CI: 2.02-10.80).

## Discussion

The results of this study demonstrate that coverage of prenatal care with at least one consultation is practically universal in Brazil, reaching levels greater than 90%, irrespective of the geographical region of the country or maternal characteristics. Nonetheless, a slightly lower coverage was observed in the North region and among women with less schooling, indigenous women, and women without a partner, probably as a result of geographical access, and cultural and social barriers. Provision of a prenatal card was a well-established practice, without regional differences, and this result was similar to that observed in a recent national study [32].

More than 80% of the women who presented with their prenatal card at the time of hospital admission for delivery had prenatal cards with the results from the first HIV serological test. This proportion was higher than that observed in the Sentinel Parturient Study of 2006 [4], when 62.8% of the pregnant women analyzed presented with an HIV serological test. Our results thus corroborate the findings from recent local studies [13,18,19,23,24], which indicated coverage close to 80%. Although a notable increase in this coverage was seen, regional differences were still observed. The South region was the only one to attain coverage greater than 90%, which is the target defined by World Health Organization (WHO) for vertical HIV transmission control programs [33], but this is still lower than the coverage of 95% proposed by PAHO for the Latin American and Caribbean region [22].

Younger women, with less schooling and who self-reported as black or mixed, who were living in the North and Northeast regions, and whose prenatal care was provided in public clinics, with a late start and an insufficient number of consultations, were the ones who presented with the lowest coverage of at least one HIV test during pregnancy. These results show the importance of an early start to prenatal care and an adequate number of consultations for undertaking routine examinations, as already demonstrated in other studies [23,34,35], thus enabling timely implementation of care protocols for preventing vertical transmission. The results also reveal inequalities in control measures that may not reach women with greater social vulnerability, i.e., younger women with less schooling and black and mixed skin color, who live in less developed regions of the country. It is important to note that this state of inequality has remained unchanged over the years, given that these results have already been observed in national studies conducted at the beginning of the 2000s [4,34-36]. Similar inequalities have been described in Africa [37].

The rate of performing a second test is still poor in the country: less than 30%, with marked regional differences. Although differences in coverage by characteristics of women have been observed, the coverage attained has been low, even among those in a better socioeconomic condition who were attended in private clinics. The Brazilian Ministry of Health introduced the second serological test for HIV in 2006 [38], as part of the prenatal care routine. The low coverage observed reveals that there have been problems in implementing this recommendation.

Brazilian Guidelines recommend that all pregnant women without any previous serological test or without serological results from the third trimester of pregnancy have a rapid HIV test performed at admission for delivery. The observed low coverage of the second serological test, both in public and in private clinics, gives rise to the need to perform testing on a greater number of women at the time of admission for delivery, thus overloading these services and making it difficult to diagnose the infection, hindering timely implementation of preventive measures against vertical transmission. Moreover, other aspects of care are also impacted, such as delaying the start of breastfeeding after delivery [39].

The prevalence of HIV infection among the pregnant women of this study (0.40%) was similar to that observed in the Sentinel Parturient Studies conducted in 2004 (0.41%) [40], 2006 (0.41%) [4], and 2010 (0.38%) [3]. However, because private clinics were not included in these Sentinel Studies, comparisons between these and our data are difficult. When the births that took place only in the public and mixed clinics of our study are analyzed separately (i.e. using the criteria of the Sentinel Studies), the estimated prevalence was significantly higher than that observed in the private clinics. This is a novel result for Brazil, and it demonstrates that there is a greater prevalence of infection among female users of clinics within the Brazilian National Health System (SUS), which is a population formed by women of poor socioeconomic condition.

The South region had the highest prevalence of HIV infection among pregnant women in this country, and the North region had the second highest prevalence, although with no significant difference in comparison to the Southeast region. Data from local studies have also shown a higher prevalence in the North (0.6%) [41] and South regions (1.7%) [42]. Studies conducted in the Southeast [24], Northeast [43] and Midwest [44,45] regions have found prevalence values not statistically different from those estimated in our study for these regions.

The results indicated a higher prevalence of HIV infection with increasing age. The decline in mortality due to AIDS in Brazil since the 2000s [3], with increasing survival among HIV-infected individuals [46,47], is one possible explanation. The high proportion of pregnant women with diagnoses of HIV infection prior to the current pregnancy, which has been reported in several national studies [11,13,14,18,19], suggests that women who have been infected for longer periods are becoming pregnant, which may also explain the increase in prevalence with age.

A higher prevalence was also observed among women with less schooling, who were single, who self-reported as black and had diagnoses of syphilis infection. Syphilis infection is a known risk factor for HIV infection [30,31], and a combined approach to these two diseases is recommended by WHO for better results in programs for preventing vertical transmission of both diseases [48].

The greater prevalence of cases among women with less than 8 years of schooling, who were single, who self-reported as black and as users of public clinics may reflect greater social vulnerability in these groups. However, the absence of other variables relating to the women’s behavior, such as sexual partnerships, drug use and access to condoms, makes it more complex to interpret the associations encountered. A study conducted in 13 Brazilian cities [49] found that there was greater use of drugs, an earlier start to sexual life, low adherence to condom use and a greater proportion of histories of STD and sexual violence among the women living with HIV/AIDS. It should be emphasized that black women, those with less schooling and those attended in public clinics, who had high odds ratios for HIV infection, were the women who presented with the lowest coverage of testing. This reveals missed opportunities for making diagnoses in the population most affected by the infection.

This study was conducted in institutions where more than 500 deliveries take place every year. It is likely that pregnant women who deliver at home or in smaller hospitals have different degrees of access to prenatal services and routine examinations. Nonetheless, given that more than 99% of deliveries in Brazil take place in hospitals, and approximately 80% in larger hospitals [25], significant changes to the results presented here would not be expected.

One limitation of the present study was that the results from the HIV tests were only ascertained among the women who presented with the prenatal card at the time of hospital admission for delivery (71.6%). However, the estimates presented here for the coverage of HIV testing during pregnancy are not thought to be overestimated, because the women who least often presented with a prenatal card were the ones with the most schooling and who were attended in private clinics, which also had higher test coverage. This less frequent presentation of the card probably resulted from the care model in the private sector, in which the same professional provides the prenatal and delivery care. Thus, it is unlikely that these women who did not present a card had done anti-HIV tests less often during pregnancy.

Another limitation of the present study was that the serological tests were not performed in a standardized manner, with use of secondary data to estimate the prevalence of HIV infection. It is possible that failures in recording the medical file data may have led to underestimation of this infection in the cases of women who did not present a prenatal card on admission for delivery.

Finally, our eligibility criteria excluded women with miscarriages. This exclusion may have introduced bias in the estimated prevalence of HIV infection in pregnancy if women with miscarriages tend to have an increased likelihood of HIV infection [50,51].

## Conclusions

The present study found a 0.40% prevalence of HIV infection among pregnant women, an almost universal coverage of antenatal care, and coverage of prenatal HIV testing of over 80%. Nevertheless, regional and social inequalities in accessing healthcare services, the low implementation of the second anti-HIV serological test, and the lower coverage of testing among women with characteristics associated with greater prevalence of HIV infection indicate shortcomings in the HIV infection control programs during pregnancy. These missed opportunities probably contributed to the high vertical transmission of HIV infection in specific group populations, despite the available resources guaranteed by public policies since the 1990s. This result stresses the importance of designing and implementing interventions targeted to the people most affected by the epidemic that would most benefit from the interventions available to prevent mother-to-child transmission of HIV infection. The monitoring of the prevalence of HIV infection among pregnant women to identify the groups most affected by the epidemic, as well as the monitoring and evaluation of antenatal care and HIV program indicators, are important tools for the development of these specific interventions.

### Endnotes section

^a^The Brazilian national HIV testing system at the time of the study [52] recommended the use of tests in two stages: a screening stage and a confirmatory stage. Rapid tests can be used in both stages if they have been validated by the National Department of STD/Aids and if they are able to detect anti-HIV-1 antibodies, including group O and HIV-2 antibodies

## Abbreviations

PAHO: Pan-American Health Organization
SUS: Brazilian National Health System
WHO: World Health Organization
